# Cyclin A as a marker for prognosis and chemotherapy response in advanced breast cancer

**DOI:** 10.1038/sj.bjc.6602735

**Published:** 2005-08-09

**Authors:** P Poikonen, J Sjöström, R-M Amini, K Villman, J Ahlgren, C Blomqvist

**Affiliations:** 1 Department of Oncology, Helsinki University Central Hospital, PO Box 180, 00029 HUS Helsinki, Finland; 2 Department of Genetics and Pathology, Uppsala University Hospital, Uppsala, Sweden; 3 Department of Oncology, Medical Centre Hospital, Örebro, Sweden; 4 Department of Oncology, Gävle Hospital and Clinical Research Centre, County of Gävleborg, Gävleborg, Sweden

**Keywords:** cyclin A, advanced breast cancer, prognosis, chemotherapy response, proliferation

## Abstract

We wanted to study cyclin A as a marker for prognosis and chemotherapy response. A total of 283 women with metastatic breast cancer were initially enrolled in a randomised multicentre trial comparing docetaxel to sequential methotrexate-fluorouracil (MF) in advanced breast cancer after anthracycline failure. Paraffin-embedded blocks of the primary tumour were available for 96 patients (34%). The proportion of cells expressing cyclin A was determined by immunohistochemistry using a mouse monoclonal antibody to human cyclin A. Response evaluation was performed according to WHO recommendations. The median cyclin A positivity of tumour cells was 14.5% (range 1.2–45.0). Cyclin A correlated statistically significantly to all other tested proliferation markers (mitotic count, histological grade and Ki-67). A high cyclin A correlated significantly to a shorter time to first relapse, risk ratio (RR) 1.94 (95% CI 1.24–3.03) and survival from diagnosis, RR 2.49 (95% CI 1.45–4.29), cutoff point for high/low proliferation group 10.5%. Cyclin A did not correlate to chemotherapy response or survival after anthracycline, docetaxel or MF therapy. Of all tumour biological factors tested (mitotic count, histological grade and Ki-67), cyclin A seemed to have the strongest prognostic value. Cyclin A is a good marker for tumour proliferation and prognosis in breast cancer. In the present study, cyclin A did not predict chemotherapy response.

Chemotherapy has an essential role in the treatment of both local and advanced breast cancer (Anonymous, 1998; Crown *et al*, 2002). Since not all patients benefit from aggressive chemotherapy, identifying factors predicting poor prognosis and chemotherapy response would be of most importance. The measurements of the proliferative cell fraction are widely used to evaluate the tumour growth rate and to establish the prognosis in cancer patients. High proliferation activity correlates strongly with poor prognosis in breast cancer, irrespective of the methodology used. According to a recent review, counting the cells in mitosis is the most reproducible and independent of the various markers for tumour proliferation, while Ki-67 labelling and cyclin A index are promising alternatives (van Diest *et al*, 2004). Mitotic count is the most important constituent of the histological grade (Genestie *et al*, 1998).

Cyclins and cyclin-dependent kinases regulate the cell cycle at specific points. Cyclin A is essential in the S phase for DNA replication, it is also involved in G2–M-phase transition (Zindy *et al*, 1992; Fan and Bertino, 1997), and is therefore one of the most useful markers signifying proliferating cells. Overexpression of cyclin A has been associated with worse prognosis for breast cancer in several studies (Bukholm *et al*, 2001; Michalides *et al*, 2002; Michels *et al*, 2002), but the results have not been consistent (Kuhling *et al*, 2003; Rudolph *et al*, 2003). Since the lack of prognostic significance of cyclin A is mainly seen in patients with lymph node-negative breast cancer, it is possible that cyclin A plays a more important role in aggressive tumours and metastatic disease (Kuhling *et al*, 2003).

Of various tumour biological factors, including different proliferation markers, only the S-phase fraction seems to be uniformly associated with response to preoperative chemotherapy, but not in metastatic disease (Sjöström, 2002). No predictive markers for the chemotherapy response are presently in use in the treatment of advanced breast cancer.

High cyclin A score predicted a better chemotherapy response and longer progression-free survival in patients with soft tissue sarcoma (Huuhtanen *et al*, 1999). To our knowledge, there are no studies of cyclin A as a predictive marker for chemotherapy response in metastatic breast cancer. The purpose of the present study is to evaluate cyclin A as a marker for tumour aggressiveness and chemotherapy response in patients with advanced breast cancer treated in a randomised trial (Sjöström *et al*, 1999).

## PATIENTS AND METHODS

### Patients

The study is based on a phase III multicentre trial, where 140 patients were randomly allocated to receive intravenous methotrexate and 5-fluorouracil, and 143 to receive intravenous docetaxel until disease progression as second-line therapy for metastatic breast cancer between December 1994 and October 1997 (Sjöström *et al*, 1999). The patients were required to have histologically proven primary breast cancer that had progressed during or after the first-line anthracycline-containing treatment for metastatic disease, or that had relapsed within 12 months after discontinuation of adjuvant anthracycline-containing regimen. The patients had to be 18–70 years old with a performance status ⩽2, and have no more than one previous chemotherapy regimen for advanced disease (multiple endocrine treatments and radiotherapy were allowed). Patients with measurable lesions or evaluable lesions were eligible. Response evaluation for docetaxel and methotrexate-fluorouracil (MF) treatment was carried out during every third course, at treatment discontinuation and every 3 months during follow-up. Anthracycline response was evaluated retrospectively from patient documents. Response evaluation was performed according to WHO recommendations (Miller *et al*, 1981). All patients with histological blocks from the primary tumour available for analysis were included in this study. All stainings were reviewed, classified and regraded by one of the authors (RMA), who did not have access to clinical data. Characteristics of the primary tumours at the time of diagnosis are shown in Table 1. Mitotic grading into three classes (1–3) was carried out according to the recommendations of Elston and Ellis (1991). Ethical approval was obtained for the randomised chemotherapy study, including subsequent studies on biological markers.

### Immunohistochemical assays

Paraffin-embedded blocks of the primary tumour were available for 96 patients (34%). All tissues had been fixed in 4% buffered formalin, processed and embedded in paraffin. From each block, 5-*μ*m-thin sections were cut on coated slides and dried overnight at 37°C. Sample deparaffination was performed in xylene and rehydration in alcohol to distilled water. Antigen demasking was carried out by heating the samples in a microwave oven (850 W) in citric acid buffer (pH 6.0) for 20 min, buffer was added in need. Treatment with 1.6% methanolperoxidase was used to inhibit endogenous peroxidase activity. For immunohistochemistry (IHC), the specimens were incubated overnight at room temperature with 1 : 100 diluted mouse monoclonal antibody to human cyclin A (Novocastra, Newcastle, UK) and for Ki-67 determination with 1 : 500 mouse anti-human monoclonal Mib-1 antibody (Immunotech, Marseille, France). The binding of the primary antibody was detected by a peroxidase-conjugated secondary antibody using an Elite ABC Kit (Vectastain, Vector Laboratories, Burlingame, CA, USA). As positive and negative controls for cyclin A stainings, we used hyperplastic tonsil tissue in cyclin A stainings. The primary antibody was omitted from negative controls.

For quantification of the immunostaining, the tumour area with the highest density of positive nuclear staining was chosen. To calculate the percentage of positively stained nuclei, an ocular grid of 100 (10 × 10) squares was used at 10 × 40 magnification. All positive nuclei from this area were counted. To estimate the negative nuclei in the same area of 100 squares, three different rows of 10 squares were counted and the mean score multiplied by 10. In the case of tumours with scarce cellularity, several fields were evaluated, and negative nuclei were counted from the whole grid area of 100 squares. The percentage of positive nuclei was estimated by dividing the number of positively stained cells by the entire number of cells in the same area.

### Statistical methods

Statistical analyses were carried out using Macintosh SPSS statistical software package. The association of TNM stage and tumour characteristics with cyclin A was tested with Spearman correlation coefficient for ordinal variables and Student's *t*-test for dichotomous variables. The association between different proliferation markers was tested by computing the nonparametric Spearman correlation coefficient. The association between treatment response and proliferation markers was measured by computing the Pearson correlation coefficient with CR classified as 4, PR as 3, NC as 2 and PD as 1, and proliferation markers as continuous variables. Overall survival (OS) and time to first relapse (TFR) curves were prepared by the Kaplan–Meier method, and prognostic variables were tested using the Cox regression analysis with proliferation markers as continuous variables. Due to multiple comparisons, a significance level of 0.01 was chosen. The impact of proliferation rate and grade on TTP and OS was tested with rates as both continuous and discrete (high/low) variables. The results were similar with these two methods. Groups with high and low proliferation rates were divided with cutoff points corresponding to Ki-67 of 25%, with the aid of linear regression analysis. A linear equation of the form *y*=*kx* was formed by regressing cyclin A and mitotic grade, respectively, against Ki-67. The cutoff point was obtained by inserting the value of 25 as *x* and solving the equation for *y*. Consequently, the cutoffs for the three different proliferation measurements were 25, 10.5% and grade 3 for Ki-67, cyclin A and mitotic grade, respectively. These cut-points identified about 65% in the high-risk groups for all markers (Table 1).

## RESULTS

The median cyclin A positivity of tumour cells was 14.5% (range 1.2–45.0). The frequency distributions of all tested proliferation markers (mitotic count, histological grade, Ki-67 and cyclin A) are presented in Table 1. Tumour characteristics at the time of diagnosis according to cyclin A positivity are presented in Table 2. All proliferation markers correlated highly statistically significantly to each other, *P*<0001. The strongest correlation was between Ki-67 and cyclin A, Spearman correlation coefficient 0.74. Spearman correlation coefficients between tumour proliferation markers are presented in Table 3.

Out of 96 patients, 58 were evaluable for response after first-line anthracycline treatment and 96 after second-line docetaxel or MF treatment (50 in the docetaxel group and 46 in the MF group). The overall response rate (CR+PR) was 47% after first-line anthracycline therapy, 46% in the docetaxel arm and 26% in the MF arm, respectively. In the parent study (*n*=283), the corresponding response rates for docetaxel and MF treatment were 42 and 21% (Sjöström *et al*, 1999). Association of the overall response rate (complete or partial response) with the investigated tumour proliferation markers is shown in Table 4. There was no significant correlation between chemotherapy response and any proliferation marker after anthracycline, docetaxel or MF treatment.

**Figure 1:**
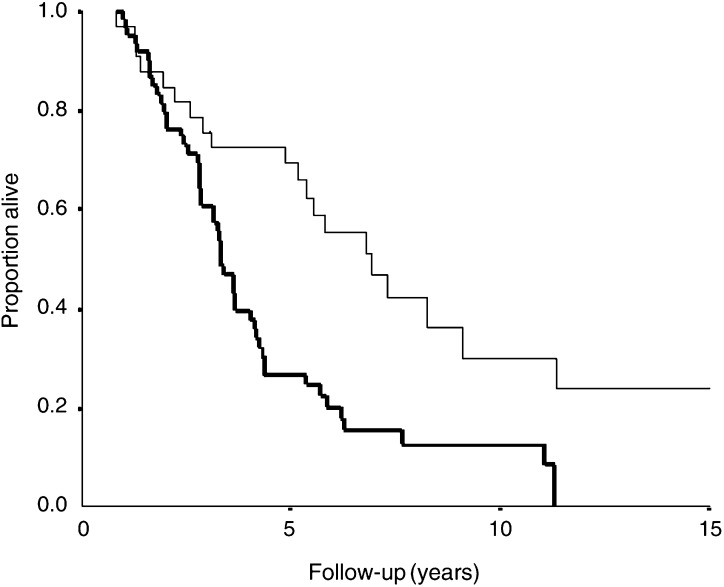
Kaplan–Meier plot for OS from diagnosis according to cyclin A. Thin line, cyclin A <10.5%; thick line, cyclin A>10.5%, *P*=0.004.

There was a significant association between a high cyclin A score and TFR, risk ratio (RR) 1.03 (95% CI 1.01–1.05), *P*=0.001 (as continuous variable) and RR 1.94 (95% CI 1.24–3.03), *P*=0.004 (as discrete variable), while the other markers showed only nonsignificant trends in the same direction. There was also a highly significant association between a high Cyclin A score and OS from diagnosis (Figure 1), RR 1.05 (95% CI 1.02–1.07), *P*<0.001 (as continuous variable) and RR 2.49 (95% CI 1.45–4.29), *P*=0.004 (as discrete variable), but not for survival from start of first- or second-line chemotherapy. For details, see the results presented in Table 5. Since the prognostic value of cyclin A seemed to depend on chemotherapy, we separately analysed the association between cyclin A and whether the patient had received adjuvant chemotherapy or not. While the prognostic impact of cyclin A on survival from diagnosis was significant for both groups, the RR was somewhat higher in patients who did not receive adjuvant chemotherapy RR 1.07 (95% CI 1.03–1.10) compared to patients who were given adjuvant chemotherapy RR 1.03 (95% CI 1.00–1.06).

## DISCUSSION

The main finding of the present study was the significant association of a high cyclin A with a short TFR and poor survival. The difference in survival from diagnosis of breast cancer was quite remarkable, despite the fact that all patients developed metastatic disease, since the patient material was recruited from a study in metastatic disease. We used a cutoff value of 10.5% for the high and low cyclin A positivity groups. This is in line with three other studies, where median values varying between 8 and 11% have been used (Michalides *et al*, 2002; Michels *et al*, 2002; Kuhling *et al*, 2003; Rudolph *et al*, 2003). In the fourth study, four different categories were used for cyclin A positivity and the median category was between 15 and 30% (Bukholm *et al*, 2001).

We found a strong correlation between cyclin A, mitotic count, tumour grade and Ki-67, which is in line with previous studies in breast cancer (Michalides *et al*, 2002; Michels *et al*, 2002). Cyclin A was the only marker that showed a statistically significant correlation to both TFR and OS; therefore, it seems to be the most useful marker of proliferation. There are some well-known problems with the use of traditional markers for proliferation in breast cancer. The duration of mitotic phase can vary and the mitotic count is not linearly correlated to proliferating cells, especially in aneuplodic tumours. Although histological grade is a well-established prognostic factor, the reproducibility of the method has been questioned (Boiesen *et al*, 2000). The flow-cytometric determination of S phase requires tumour volumes larger than immunohistochemical methods, and there is also pronounced intratumoral heterogenity in S phase (van Diest *et al*, 2004). Moreover, fresh frozen tissue is needed for S-phase analysis and the costs for flow cytometry are higher than for IHC. Cyclin A is expressed during the late S, G2 and M phases, and it is therefore a useful marker for proliferating cells (Zindy *et al*, 1992; Fan and Bertino, 1997). Cyclin A can be analysed on paraffin-embedded tissue with the immunohistochemial technique, which is an advantage both for practical and economical reasons.

An association between a high cyclin A expression and poor prognosis has previously been described in three studies, all executed after primary therapy for breast cancer (Bukholm *et al*, 2001; Michalides *et al*, 2002; Michels *et al*, 2002). A fourth study, however, in node-negative patients showed no correlation between cyclin A and prognosis (Kuhling *et al*, 2003; Rudolph *et al*, 2003). Several studies have revealed an association between a high proliferation index measured by other means and favourable chemotherapy response. In the preoperative setting, most studies have shown that a higher proliferation index is associated to a higher response rate of anthracycline-based chemotherapy, while the results in metastatic breast cancer are less consistent (Sjöström, 2002). There are data also from other malignancies, indicating that a higher proliferation rate correlates to a better chemotherapy response (Joensuu *et al*, 1994; Hahka-Kemppinen *et al*, 1997; Huuhtanen *et al*, 1999). We are aware of no clinical data on cyclin A as a predictive factor for chemotherapy in breast cancer. A preclinical study by Volm showed that breast cancer cell lines with high cyclin A were significantly more sensitive to doxorubicin than cell lines with low cyclin A activity (Volm *et al*, 1997).

Anthracyclines are active throughout the cell cycle, but the effects are most pronounced for cells in S or G2 phase. The antimetabolites methotrexate and 5-fluorouracil as antimetabolites mainly inhibit the cell proliferation in the S phase, and docetaxel, which is a microtubulin stabiliser, exerts its cytotoxic effect in the G2M phase. Since cyclin A is active and detectable from the beginning of the S phase to the beginning of mitosis, it should theoretically label the proportion of cells that are sensitive to chemotherapeutic drugs used in our study. Despite this, no correlation between cyclin A activity and response rate, TTP and OS calculated from the start of chemotherapy was found. A previous study from a randomised trial comparing adjuvant chemotherapy (cyclophosphamide, methotrexate and 5-fluorouracil) to radiotherapy in primary breast cancer showed that the benefit from chemotherapy was more pronounced among patients with tumours with a high S-phase fraction (Stal *et al*, 1994). Thus, the strong contrast between the adverse prognostic impact of a high proliferation rate from diagnosis of the disease, but not after start of aggressive chemotherapy, may indicate that chemotherapy has improved the course of the disease, especially in highly proliferating tumours.

We conclude that cyclin A seems to be the strongest prognostic factor in a panel of proliferation markers including Ki-67 and mitotic count in metastatic breast cancer. None of the proliferation markers predicted chemotherapy response to the three regimens in the study.

## Figures and Tables

**Table 1:** Characteristics of the primary tumours at the time of diagnosis and time to first relapse (TFR) of the 96 investigated patients

**Factor**	**No. of patients (%)**	**Median (range)**
*Histology*		
Ductal	94 (98)	
Lobular	2 (2)	

*Oestrogen receptor status*		
Positive	48 (50)	
Negative	39 (41)	
Unknown	9 (9)	

*Mitotic count*		
1	12 (13)	
2	29 (30)	
3	55 (57)	

*Tumour grade*		
1	1 (1)	
2	32 (33)	
3	63 (66)	

*KI-67*		
<25%	29 (30)	38 (10–90)
⩾25%	67 (70)	

*Cyclin A*		
<10.5%	33 (34)	14.5 (1.2–45)
⩾10.5%	63 (66)	

TFR (years)		1.57 (0–22.8)

**Table 2:** Tumour characteristics at the time of diagnosis according to cyclin A positivity

	**Cyclin A<10.5%**	**Cyclin A>10.5%**	
**Tumour characteristic**	**No. of patients (%)**	**No. of patients (%)**	***P*-value**
*Tumour size*			
0–2 cm	10 (30)	14 (22)	
2.1–5 cm	12 (36)	29 (46)	
>5 cm	4 (12)	9 (14)	
Direct extension to skin or chest wall	2 (6)	4 (6)	NS
Unknown	5 (15)	7 (11)	

*Nodal status*			
Negative	12 (36)	19 (30)	
Positive	18 (55)	36 (57)	
Fixed lymph nodes	2 (6)	7 (11)	
Unknown	1 (3)	1 (2)	NS

*Primary metastatic disease*			
No	23 (70)	51 (81)	
Yes	6 (18)	7 (11)	
Unknown	4 (12)	5 (8)	NS

*Hormone receptor status*			
ER+	10 (30)	29 (46)	
ER−	21 (64)	27 (43)	
Unknown	2 (6)	7 (11)	*P*=0.003

*Histology*			
Ductal	31 (94)	63 (100)	
Lobular	2 (6)		

*c-erb-B2*			
Low	5 (15)	29 (46)	
Intermediate	3 (9)	16 (25)	
High	11 (33)	18 (28)	NS

*Grade*			
1	3 (9)	1 (2)	
2	17 (52)	13 (21)	
3	13 (39)	49 (78)	*P*<0.001

**Table 3:** Spearman correlation coefficient and significance between the investigated tumour biological factor

	**Grade**	**KI-67**	**Cyclin A**
Mitotic count	0.82	0.39	0.39
Grade		0.36	0.40
KI-67			0.74

*P*-value 0.001 for all comparisons.

**Table 4:** Association of the overall response rate (complete or partial response) to different chemotherapy with the investigated tumour biological factors in patients with evaluable response to treatment

**Variable**	**Response % to anthracycline treatment (*n*)**	***P*-value^a^**	**Response % to docetaxel treatment (*n*)**	***P*-value^a^**	**Response % to MF treatment (*n*)**	***P*-value^a^**
All tumours	47 (58)		46 (48)		26 (46)	

*Mitotic count*						
1	56 (5/9)		60 (3/5)		43 (3/7)	
2	38 (6/16)		39 (7/18)		36 (4/11)	
3	49 (16/33)	0.89	48 (13/27)	0.94	18 (5/28)	0.55

*Tumour grade*						
1	100 (1/1)					
2	29 (5/17)		47 (9/19)		46 (6/13)	
3	53 (21/40)	0.32	47 (14/30)	0.78	18 (6/33)	0.16

*KI-67*						
<25%	41(7/17)		43 (6/14)		47 (7/15)	
⩾25%	49 (20/41)	0.27	47 (17/36)	0.97	16 (5/31)	0.18

*Cyclin A*						
<10.5%	42 (8/19)		59 (10/17)		44 (7/10)	
⩾10.5%	49 (19/39)	0.11	39 (13/33)	0.55	17 (5/30)	0.26

a *P*-value for the Pearson correlation coefficient.

**Table 5:** Cox regression analysis according to tumour biological factor (as discrete, high/low variable) and different chemotherapy regimen

**Risk ratio (95% CI)**				
	**Grade**	**Mitotic count**	**KI-67**	**Cyclin A**
TFR	1.50 (0.97–2.32)	1.29 (0.86–1.95)	1.36 (0.88–2.11)	1.94 (1.24–3.03)
OSdg	1.95 (1.16–3.27)	1.62 (0.99–2.65)	1.61 (0.95–2.70)	2.49 (1.45–4.29)

*Efficacy parameters after anthracycline treatment*				
TTPa	0.46 (0.22–0.97)	0.82 (0.42–1.60)	0.83 (0.40–1.71)	0.63 (0.31–1.27)
OSa	1.11 (0.56–2.20)	1.01 (0.56–1.91)	1.28 (0.64–2.55)	1.34 (0.67–2.68)

*Efficacy parameters after docetaxel or MF treatment*				
TTPdoc	1.02 (0.56–1.87)	0.83 (0.46–1.51)	0.63 (0.32–1.24)	1.32 (0.70–2.48)
OSdoc	1.46 (0.75–2.86)	0.65 (0.61–2.21)	0.85 (0.41–1.76)	1.90 (0.90–4.06)
TTPmf	1.30 (0.66–2.54)	1.08 (0.58–2.00)	1.19 (0.62–2.26)	1.20 (0.64–2.26)
OSmf	1.25 (0.56–2.83)	0.92 (0.46–2.02)	1.14 (0.53–2.46)	1.01 (0.48–2.13)

TFR: time to first relapse, TTPa: time to progression after anthracycline therapy, OSa: overall survival after anthracycline therapy, TTPdoc: time to progression after docetaxel therapy, OSdg: overall survival from diagnosis, OSdoc: overall survival after docetaxel therapy, MF: methotrexate fluorouracil, TTPmf: time to progression after methotrexate fluorouracil therapy, OSmf: overall survival after methotrexate fluorouracil therapy.
